# Dementia Caregiver Satisfaction and Utilization of a Telehealth App: Results From the FamTechCare Trial

**DOI:** 10.1093/geroni/igaa057.487

**Published:** 2020-12-16

**Authors:** Kristine Williams, Clarissa Shaw, Yelena Perkhounkova, Maria Hein, Carissa Coleman

**Affiliations:** 1 University of Kansas, Lawrence, Kansas, United States; 2 University of Iowa, Iowa City, Iowa, United States; 3 University of Kansas, Kansas City, Kansas, United States

## Abstract

Technology can enhance support for families caring for persons with dementia but must be acceptable to be adopted. In the FamTechCare trial, caregivers used an app to videorecord care encounters that were reviewed by an expert panel who provided tailored feedback. The intervention reduced caregiver depression and improved caregiver competence. This mixed methods study reports caregiver satisfaction and utilization of the intervention and evaluation of the intervention by the expert panel. A convergent parallel mixed methods design was used to evaluate the satisfaction, usability, and feasibility of the intervention. Caregiver-person with dementia dyads were randomized to the FamTechCare video support or telephone attention control support groups. Caregivers completed a satisfaction survey at the completion of the 3-month trial. The number and duration of videos submitted and calls received by caregivers were used to evaluate utilization. Relationships between participant characteristics and satisfaction and utilization were evaluated. Feasibility and future directions for the intervention were assessed through interviews with the expert panel. The majority of caregivers in both groups reported benefits from participation. More FamTechCare caregivers found the interventionist support to be helpful (p=.001) and effective (p=.020) compared to attention control caregivers. FamTechCare caregivers of persons with more severe dementia were more likely to report that video recording intruded on their privacy (p=.050). Age, gender, education, dyad relationship, rural status, and type and severity of dementia were not associated with ratings of acceptability, ease of use, or intervention utilization. The expert panel rated FamTechCare as useful and identified adaptations to enhance feasibility.

